# Notes and News

**Published:** 1895-12-28

**Authors:** 


### Notes and News.

(Collected and Communicated.)

The sixth International Congress of Otology will be held in London in 1899.

Our Association for the Suppression of Street Noises has now a parallel in New York, where an Anti-Noise League has been formed. We wish the league may secure more successful results than have attended the older society.

Mr. Cole, the excellent secretary of the West of England Eye Infirmary at Exeter, who has held the office for thirty-four years, has resigned, and is succeeded by Mr. E. W. Toby, who has acted as his assistant for some years.

It is the custom to celebrate the Hospital Saturday Fund collection at Croydon by an annual dinner in which those most actively interested in the institution join. At the dinner held last week £505 was handed over to the Croydon General Hospital, the result of this year's collection.

An effort is being made by the Council of the Printers' Almshouses to raise a sum of £10,000 to permanently endow the almshouses. New buildings were erected at Wood Green not long ago, and the institution is patronised by the Queen and the Prince of Wales.

The committee of the After-Care Association for Poor Persons Discharged Recovered from Asylums for the Insane earnestly appeal for funds to enable them to carry on and extend their efforts. The objects of the association are to assist cases (male and female) on their discharge from asylums for the insane. Subscriptions and donations thankfully received by the secretary, Mr. H. Thornhill Roxby, Church House, Dean's Yard, Westminster; or they may be paid into the account of the "After-Care Association," Union Bank of London (Regent Street Branch), Argyll Place, W.

The Metropolitan Asylums Board have arranged to throw open the Gore Farm Hospital to scarlet fever patients this week.

Sir W. H. Broadbent has accepted the post of consulting physician at the West-End Hospital for Nervous Diseases, vacant by the death of Dr. Bristowe.

A meeting was held recently at Cheltenham, under the presidency of the Mayoress, in aid of new buildings for the children's home and hospital. The present institution is unsuitably situated, and has no garden. It was agreed to commence a collection in aid of the building fund, and nineteen ladies have already undertaken to collect 1,000 shillings each towards it.

Dr. Penny, house surgeon to the North Devon Infirmary, Exeter, has been elected secretary of the institution. The meeting held for the purpose of the election was a stormy one, as some opposition was raised against Dr. Penny's candidature, owing to his holding the post of house surgeon. The infirmary possesses what is termed an acting secretary as well, but we believe it is very unusual for any hospital to require more of its medical officers than an attention to the medical duties of the post, beyond the attendance at committee meetings.

For the benefit of those travelling southwards, we mention that a new route to Egypt, *viâ* Ostend. Vienna, and Trieste, is now arranged for. The International Sleeping Car Company announce the organisation of a new weekly service *de luxe* from London, *viâ* Ostend and Vienna to Trieste, for Alexandria. The train will be composed exclusively of sleeping and dining cars and through baggage vans, and will run through from Ostend to Trieste without change. At that port it will connect with one of the large and well-appointed steamers of the Austrian-Lloyd Company. The service will leave London every Sunday at ten a.m., Trieste every Tuesday, and will arrive at Alexandria every Saturday at four p.m. On the return journey the steamer will leave Alexandria every Saturday at nine a.m. The train *de luxe* will leave Trieste every Wednesday on arrival of the steamer, and will arrive in Ostend and London every Friday.

The United States Government have been trying experiments in connection with their Army Commissariat, which are both instructive and useful, though they have proved very trying to the subjects of these experiments. The troops of the American Army are especially exposed to the difficulty of carrying or obtaining food on long marches in their country. An attempt, therefore, to supply condensed forms of food was made. Coffee, soup, bread, and bacon were all treated to the condensing process, and a company of soldiers thus supplied started on a ten days' march. Four days' experience of these rations ended in reducing the company to a condition of ill-health, and the march had to be abandoned, several of the men being confined to the hospital for some days. The result of these and other experiments has proved that quantity in bulk is as essential a condition of human food as the quality of the ingredients. No better diet than hard tack, coffee and bacon, in emergencies, has yet been essayed n the United States Army.

